# Employment status and changes in working career in relation to asthma: a cross-sectional survey

**DOI:** 10.1186/s12995-018-0189-6

**Published:** 2018-02-14

**Authors:** Saara Taponen, Lauri Lehtimäki, Kirsi Karvala, Ritva Luukkonen, Jukka Uitti

**Affiliations:** 1 Finla Occupational Health, Satakunnankatu 18 B, 33210 Tampere, Finland; 2 Allergy Centre, Tampere University Hospital, PO Box 2000, 33521 Tampere, Finland; 3 Faculty of Medicine and Life Sciences, University of Tampere, 33014 Tampere, Finland; 4 Finnish Institute of Occupational Health, PO Box 40, 00251 Helsinki, Finland; 5 Clinicum, Faculty of Medicine, University of Helsinki, PO Box 63, 00014 Helsinki, Finland

**Keywords:** Work ability, Asthma, Job change, Career change

## Abstract

**Background:**

Asthmatics confront inconveniences in working life that make it more difficult to pursue a sustainable career, such as unemployment and work disability. Ways of dealing with these inconveniences may be career changes. More needs to be known about the backgrounds and consequences of career changes among asthmatics, especially their relation to asthma or a change in asthma symptoms. The aim of this study was to compare earlier career changes of adults with asthma who are working full time to those who have drifted away from active working life because of work disability, unemployment or early retirement. The frequency of having changed tasks, work place or occupation, whether the changes had been driven by asthma and furthermore, whether the changes had affected their asthma symptoms were investigated.

**Methods:**

In this population-based survey study, all patients with reimbursement rights for asthma aged 20–65 years in the city of Tampere (total population 190,000), Finland (*n* = 2613) were recruited. The questionnaire was sent in October 2000 and the response rate was 79%. The questionnaire included questions e.g. on changing tasks, work place and occupation, whether these changes were driven by asthma or associated with change of asthma symptoms. The respondents were divided into four groups: working full-time, work disability, unemployed and retired due to age. We applied ANOVA with Dunnet’s post-test (variances were not equal between the groups) for a continued variable age and Chi-squared tests for categorical variables. Logistic regression models were built using unemployed vs. full-time work or work disability vs. full-time work as an outcome variable. A *p*-value of <.05 was considered statistically significant.

**Results:**

Adults with asthma working full time had more often made changes in their career, but not as often driven by asthma as those with current work disability. The reason for changing work place compared to full-time workers (24.9%) was more often mainly or partly due to asthma among those with work disability (47.9%, *p* < 0.001) and the unemployed (43.3%, *p* = 0.006). Of those who made career changes because of asthma, a major proportion (over 67%) reported relief in asthma symptoms. Changing tasks (OR 5.8, 95% CI 1.9–18.0, for unemployment vs. full-time work), work place (OR 2.8, 95% CI 1.1–7.0, for work disability vs. full-time work and OR 2.6, 95% CI 1.3–5.4, for unemployment vs. full-time work) or occupation (OR 2.7, 95% CI 1.2–6.0, for unemployment vs. full-time work) mainly because of asthma was associated with an elevated risk for undesirable employment status even after adjusting for age, gender, smoking and professional status.

**Conclusions:**

Career changes that were made mainly because of asthma were associated with undesirable employment status in this study. However, asthma symptoms were relieved after career changes especially among those who reported asthma to be the reason for the change. In addition to proper treatment and counselling of asthma patients towards applicable area of work or study, it may be beneficial to support early career changes in maintaining sustainable working careers among adults with asthma.

## Background

Sustainable work has been set as a main goal by European Union countries, meaning that ‘living and working conditions are such that they support people in engaging and remaining in work throughout an extended working life‘[1]. For an employee with asthma, maintaining a sustainable working career may be a significant challenge. Selective exclusion from work as a consequence of asthma is observed in childhood asthmatics already at the beginning of their working life and for current adult-onset asthmatics at the end of their working life [2]. Throughout working life, adults with asthma commonly change jobs due to respiratory problems at work [3], they often deal with sickness absence [4], delayed return to work [5] or complete cessation of employment due to respiratory problems [6]. When work ability is reduced due to other health conditions, having asthma makes it even more difficult to return to work. For example, a large cohort study by Ervasti et al. [7] showed that return to work after sickness absence because of depression is delayed in the presence of asthma. In a study of public sector employees, asthma increased the risk of all-cause long-term work disability 1.8-fold compared to controls with no asthma. [8]

Work-related asthma symptoms may have a considerable socio-economic impact, job change or work loss due to asthma [9]. Work-exacerbated asthma (WEA) is common (medium prevalence 21.5% among adults with asthma) and occasionally supporting job change is needed to manage WEA when reducing exposures and optimizing treatment is not enough [10].

As these earlier studies have shown, adults with asthma may struggle with inconveniences in working life that make it more difficult to pursue a sustainable career. Changing jobs is common among employees with asthma and it may be one way of coping in working life with asthma. However, more needs to be known about the backgrounds and consequences of changing jobs among adults with asthma in order to focus on effective support of their working careers.

We have found in our earlier study that among adults with asthma, full-time workers are on average younger, more frequently nonmanual workers, they smoke less and have less asthma symptoms both at work and at leisure time despite of using less asthma medication than those who are unemployed, have work disability or are retired [11]. Full-time work was interpreted as an indicator of successful coping in working life. Now we wanted to analyze further, if changes in working career in relation to asthma could explain the better success in coping in working life of some adults with asthma.

The aim of this study was to compare earlier career changes of adults with asthma who are working full time to those who have drifted away from active working life because of work disability, unemployment or early retirement. We studied the frequency of having changed tasks, work place or occupation, whether the changes had been driven by asthma and furthermore, whether the changes had affected their asthma symptoms.

## Methods

This cross-sectional survey study recruited all patients with reimbursement rights for asthma medication (*n* = 2613) aged 20–65 years and living in the city of Tampere (total population 190,000), Finland. The cases were identified from the Medication Reimbursement Register of the Finnish Social Insurance Institution. All those who had been granted special reimbursement rights for asthma medication until the end of 1997 and were alive in October 2000 were selected. To be granted reimbursement rights by the Finnish Social Insurance Institution, the disease must fulfill the diagnostic and severity criteria of asthma, including objective data of reversible/variable bronchial obstruction and a need for regular treatment with inhaled glucocorticoids for at least 6 months after the diagnosis. Among those granted special reimbursement rights for asthma medication, the reliability of the asthma diagnosis is high [12]. The questionnaire was sent in October 2000 and the response rate was 79%. Ninety-eight subjects were excluded from the analyses because they responded not having been diagnosed for asthma indeed by a physician (some individuals with reimbursement may have had e.g. asthma-COPD overlap) or because the employment status information was missing. For this study, the respondents were divided into five groups according to their working life status. There were 967 subjects working full-time, 197 unemployed subjects, 334 subjects with work disability (including all-cause sickness absence, disability pension, and disability pension applied but not yet granted) and 159 subjects retired due to age (in Finland, age-related pension starts at age of approximately 60–68 years depending on occupation and personal preferences). The fifth group consisting of subjects outside of full-time work for other reasons (e.g. housewives, students, part-time workers, maternity leave, etc.) were excluded from this study (*n* = 309).

### Questionnaire

The self-administered questionnaire included questions on employment status, changes of work tasks, work places and occupation, whether the changes were driven by asthma and whether they affected asthma symptoms (Table 2).

### Statistical analyses

We compared career changes in relation to asthma between different groups of working life status and we were especially interested whether full-time workers differ from the subjects in other three groups (unemployed, those with work disability and those retired). Our data set consisted of both continuous and categorical variables. When comparing the differences between groups we applied ANOVA with Dunnet’s post-test (variances were not equal between the groups) for a continued variable age and Chi-squared tests for categorical variables. Among those who had made career changes we were interested whether asthma was mainly or partly the reason for those changes (“mainly” or “partly” or “none”) and how a possible change affected asthma symptoms (“aggravated” or “no change” or “relieved”). The results are presented in Table 2. We computed the same results by limiting the data for those who had answered that asthma was mainly or partly the reason for career changes. Those results are presented in Table 3.

After these preliminary studies we built logistic regression models using unemployed vs. full-time work or work disability vs. full-time work as an outcome variable. Change of tasks within the same employer, change of work place, and change of occupation and whether the changes were driven by asthma (“mainly” or “partly” vs. “none”), and relief or aggravation of asthma symptoms after the changes were used as independent variables one at a time. In our data there were only very few persons who had answered that a career change aggravated the symptoms. Therefore, we combined the categories “aggravated” and “no change” before modeling. Our model building strategy was as follows: at first we estimated crude models and then adjusted models with age, gender, smoking, and professional status. The odds ratios (OR) with 95% confidence intervals (95% CI) are presented in Table 4. A *p*-value of <.05 was considered statistically significant. All analyses were carried out using SPSS (version 24) program.

## Results

Characteristics of the five groups are presented Table 1 [11].

**Table 1:** Characteristics according to working life status

	Full-time work *n* = 967	Unemployed *n* = 197	Work disability *n* = 334	Retired *n* = 159	*p*-value 1 vs. 2	*p*-value 1 vs. 3	*p*-value 1 vs. 4
Age, mean years (SD)	44.1 (10.2)	46.2 (11.0)	57.9 (8.2)	62.9 (3.8)	0.064	<.001	<.001
Gender							
Women	568 (58.7)	136 (69.0)	206 (61.7)	108 (67.9)	0.007	0.346	0.028
Men	399 (41.3)	61 (31.0)	128 (38.3)	51 (32.1)			
Smoking status							
Never smoker	432 (45.0)	70 (36.1)	132 (40.0)	92 (58.2)	0.004	0.002	<.001
Ex-smoker	217 (22.6)	40 (20.6)	98 (29.7)	38 (24.1)			
Current smoker	155 (16.1)	52 (26.8)	66 (20.0)	20 (12.7)			
Occasional smoker	157 (16.3)	32 (16.5)	34 (10.3)	8 (5.1)			
Professional status							
Self-employed	98 (10.2)	10 (5.2)	25 (8.0)	7 (4.6)	<.001	<.001	0.001
Upper level nonmanual worker	199 (20.7)	11 (5.8)	21 (6.7)	24 (15.9)			
Lower level nonmanual worker	284 (29.5)	50 (26.2)	59 (18.8)	35 (23.2)			
Manual worker	353 (36.7)	113 (59.2)	195 (62.1)	79 (52.3)			
Working from home, student, other	28 (2.9)	7 (3.7)	14 (4.5)	6 (4.0)			

Data is presented as n (%) unless otherwise stated

Work and career changes in different employment status groups are presented in Table 2.

**Table 2:** Earlier changes in working career according to current employment status

			Full-time workers *n* = 951 – 954	Unemployed *n* = 176	Work disability *n* = 246 – 256	Retired *n* = 139	*p*-value 1 vs.2	*p*-value 1 vs. 3	*p*-value 1 vs. 4
Change of tasks within the same employer after asthma diagnosis		No Yes	755 (79.4) 196 (20.6)	141 (80.1) 35 (19.9)	198 (77.3) 58 (22.7)	110 (79.1) 29 (20.9)	0.827	0.476	0.945
	If yes, was asthma the reason for changing tasks?	Mainly	43 (22.3)	13 (39.4)	19 (33.9)	12 (44.4)	<.001	0.001	0.008
		Partly	41 (21.2)	14 (42.4)	21 (37.5)	8 (29.6)			
		No	109 (56.5)	6 (18.2)	16 (28.6)	7 (25.9)			
	If yes, how did change of tasks affect asthma symptoms?	Aggravated	7 (3.9)	3 (9.4)	8 (16.0)	1 (4.2)	0.073	0.001	0.211
		No change	86 (48.0)	9 (28.1)	13 (26.0)	7 (29.2)			
		Relieved	86 (48.0)	20 (62.5)	29 (58.0)	16 (66.7)			
Change of work place after asthma diagnosis		No Yes	373 (60.1) 381 (39.9)	107 (60.1) 71 (39.9)	202 (80.2) 50 (19.8)	119 (85.6) 20 (14.4)	0.991	<.001	<.001
	If yes, was asthma the reason for changing work place?	Mainly Partly No	52 (13.8) 42 (11.1) 283 (75.1)	18 (26.9) 11 (16.4) 38 (56.7)	17 (35.4) 6 (12.5) 25 (52.1)	5 (27.8) 2 (11.1) 11 (61.1)	0.006	<.001	0.249
	If yes, how did change of work place affect asthma symptoms?	Aggravated	8 (2.4)	2 (3.4)	3 (7.0)	1 (5.9)	0.052	0.058	0.361
		No change	206 (62.6)	27 (45.8)	20 (46.5)	8 (47.1)			
		Relieved	115 (35.0)	30 (50.8)	20 (46.5)	8 (47.1)			
Change of occupation after asthma diagnosis		No Yes	708 (74.2) 246 (25.8)	122 (70.5) 51 (29.5)	202 (81.1) 47 (18.9)	122 (89.1) 15 (10.9)	0.310	0.024	<.001
	If yes, was asthma the reason for changing occupation?	Mainly	61 (23.3)	20 (35.7)	22 (41.5)	10 (45.5)	0.086	0.020	0.069
		Partly	54 (20.6)	13 (23.2)	10 (18.9)	3 (13.6)			
		No	147 (56.1)	23 (41.1)	21 (39.6)	9 (40.9)			
	If yes, how did change of occupation affect asthma symptoms?	Aggravated	6 (2.7)	2 (4.4)	4 (9.5)	1 (6.7)	0.781	0.015	0.004
		No change	100 (45.7)	20 (44.4)	11 (26.2)	1 (6.7)			
		Relieved	113 (51.6)	23 (51.1)	27 (64.3)	13 (86.7)			

Data is presented as n (%)

### Changing tasks

Changing tasks within the same employer after asthma diagnosis was similar in all groups (about 20% had changed tasks). 4.5% of full-time workers, 7.5% of the unemployed, 7.6% of the work disability group and 8.8% of the retired had changed tasks mainly because of asthma. Of those who had changed tasks, 43.5% of full-time workers compared to over 71% of all other groups reported that asthma was mainly or partly the reason for the change. It was most common to have been done the change mainly because of asthma in the retired group (44.4%), and more common in the work disability group (33.9%) and the unemployed group (39.4%) than in the full-time working group (22.3%).

### Changing work place

The change of work place after asthma diagnosis was more common among full-time workers (39.9%) and the unemployed (39.9%) compared to the work disability group (19.8%) and to the retired group (14.4%). 5.5% of full-time workers, 10.3% of the unemployed, 6.8% of the work disability group and 3.7% of the retired had changed work place mainly because of asthma. The reason for changing work place was more often mainly or partly due to asthma among those with work disability (47.9%) and the unemployed (43.3%) compared to full-time workers (24.9%).

### Changing occupation

Changing occupation after asthma diagnosis was less common than changing work place. Full-time workers reported changing occupation more frequently (25.8%) than subjects with work disability (18.9%) or the retired (10.9%). 6.4% of full-time workers, 11.6% of the unemployed 8.8% of the work disability group and 7.3% of the retired had changed occupation mainly because of asthma. Making the change because of asthma was overall more common in changing occupation than in changing work place. Of those who had changed occupation, making the change mainly or partly because of asthma was more likely among those with work disability (60.4%) than among full-time workers (43.9%).

### Career changes and asthma symptoms

After all work and career changes, it was common to report that the symptoms were relieved, especially after occupation change (> 50%). Noticeable is that in the work disability group, aggravation of symptoms after all career changes was more frequently reported than in other groups.

### Retirement

Of those who had retired after asthma diagnosis (80.6% of the work disability group and 72.2% of the retired group), a significantly greater proportion of those retired because of work disability (39.4%) compared to those retired for old age (9.3%) reported that asthma was the main reason for their retirement. Among those retired for old age, 53.7% reported that asthma was not the reason for their retirement whereas 23.6% of those retired for work disability reported the same. However, after retirement, asthma symptoms were alleviated in both groups (60.7% in work disability group and 66.3% in retired group).

Changes of asthma symptoms in those adults with asthma who reported making work or career changes because of asthma are presented in Table 3. Among those who made the change due to asthma, relief of asthma symptoms after changing tasks was significantly more common among full-time workers than among the work disability group. Of those who changed work place because of asthma, relief of asthma symptoms was more common (85–90%) in all other groups than the work disability group, of whom 68% reported relief of asthma symptoms. Of those who had changed occupation, relief of asthma symptoms was reported frequently (90–100%) among full-time workers and the retired and less frequently (72–76%) among the unemployed and the work disability group.

**Table 3:** Asthma symptom changes in those who had made career changes because of asthma

			Full-time workers (1) n = 951–954	Unemployed (2) n = 176	Work disability (3) n = 246–256	Retired (4) n = 139	*p*-value 1 vs.2	*p*-value 1 vs. 3	*p*-value 1 vs. 4
Change of tasks within the same employer after asthma diagnosis									
	Was asthma the reason for changing tasks?	Mainly	43 (51.2)	13 (48.1)	19 (47.5)	12 (60.0)	<.783	<.701	0.478
		Partly	41 (48.8)	14 (51.9)	21 (52.5)	8 (40.0)			
	How did change of tasks affect asthma symptoms?	Aggravated	2 (2.5)	2 (8.0)	3 (8.3)	0 (0.0)	0.182	0.036	0.978
		No change	11 (13.6)	5 (20.0)	9 (25.0)	3 (15.8)			
		Relieved	68 (84.0)	18 (72.0)	24 (66.7)	16 (84.2)			
Change of work place after asthma diagnosis									
	Was asthma the reason for changing work place?	Mainly	52 (55.3)	18 (62.1)	17 (73.9)	5 (71.4)	0.521	0.104	0.465
		Partly	42 (44.7)	11 (37.9)	6 (26.1)	2 (28.6)			
	How did change of work place affect asthma symptoms?	Aggravated	1 (1.1)	0 (0.0)	0 (0.0)	0 (0.0)	0.497	0.017	0.549
		No change	8 (9.0)	4 (14.8)	7 (31.8)	1 (14.3)			
		Relieved	80 (89.9)	23 (85.2)	15 (68.2)	86 (85.7)			
Change of occupation after asthma diagnosis									
	Was asthma the reason for changing occupation?	Mainly	61 (53.5)	20 (60.6)	22 (71.0)	10 (76.9)	0.470	0.082	0.107
		Partly	53 (46.5)	13 (39.4)	9 (29.0)	3 (23.1)			
	How did change of occupation affect asthma symptoms?	Aggravated	0 (0.0)	0 (0.0)	1 (3.4)	0 (0.0)	0.013	0.042	0.600
		No change	10 (9.7)	8 (27.6)	6 (20.7)	0 (0.0)			
		Relieved	93 (90.3)	21 (72.4)	22 (75.9)	13 (100)			

Data is presented as n (%)

### Associations between employment status and changes in working career and their relation to asthma

Associations between employment status and earlier career changes: changing tasks, work place or occupation, and associations between employment status and whether the career change was made due to asthma or was associated with a change in asthma symptoms, were calculated using logistic regression models and are presented in Table 4. After adjusting for age, gender, smoking and professional status, those who had changed tasks mainly because of asthma, were more likely to be unemployed than work full-time (OR 5.8, 95% CI 1.9–18.0) but between work disability group and full-time working group the difference was not statistically significant. Those who had changed work place mainly because of asthma were more likely to have work disability than work full-time (OR 2.8, 95% CI 1.1–7.0) and more likely to be unemployed than full-time workers (OR 2.6, 95% CI 1.3–5.4). Those who had changed occupation mainly because of asthma, were more likely to be unemployed than full-time workers (OR 2.7, 95% CI 1.2–6.0) but between work disability group and full-time working group the difference was not statistically significant. Relief or aggravation of asthma symptoms after changing tasks, work place or occupation was not associated with employment status.

**Table 4:** Associations between changes in working career and employment status. Crude logistic regression models and models adjusted for age, gender, smoking, and professional status

		Unemployed vs. full-time work		Work disability vs. full-time work	
		Crude OR (95% CI)	Adjusted OR (95% CI)	Crude OR (95% CI)	Adjusted OR (95% CI)
Was asthma the reason for changing tasks?	Mainly vs. No Partly vs. No	7.8 (2.7–22.6) 6.1 (2.2–17.4)	5.8 (1.9–18.0) 4.7 (1.6–14.4)	6.2 (2.5–15.2) 5.4 (2.3–12.6)	2.5 (0.8–7.7) 2.9 (1.0–8.4)
How did change of tasks affect asthma symptoms?	Aggravated or No change vs. Relieved	0.4 (0.2–1.0)	0.6 (0.3–1.6)	0.7 (0.4–1.4)	1.6 (0.7–3.9)
Was asthma the reason for changing work place?	Mainly vs. No Partly vs. No	3.1 (1.6–6.0) 2.1 (1.0–4.5)	2.6 (1.3–5.4) 1.9 (0.9–4.3)	3.6 (1.7–7.7) 1.5 (0.5–4.1)	2.8 (1.1–7.0) 1.8 (0.6–5.8)
How did change of work place affect asthma symptoms?	Aggravated or No change vs. Relieved	0.5 (0.3–0.8)	0.6 (0.3–1.1)	0.7 (0.3–1.4)	1.2 (0.5–2.8)
Was asthma the reason for changing occupation?	Mainly vs. No Partly vs. No	3.1 (1.5–6.6) 2.0 (0.9–4.5)	2.7 (1.2–6.0) 1.7 (0.7–4.2)	2.8 (1.3–5.9) 1.4 (0.6–3.4)	1.6 (0.6–3.9) 1.4 (0.5–3.8)
How did change of occupation affect asthma symptoms?	Aggravated or No change vs. Relieved	0.9 (0.4–1.7)	1.0 (0.5–2.1)	0.6 (0.3–1.1)	0.9 (0.4–2.0)

## Discussion

Our cross-sectional study among adults with asthma shows that those who are currently full-time workers have made changes more often in their career than adults with asthma with current work disability. However, asthma was more seldom the reason for these changes among full-time workers than among those with work disability. Particularly among those who made one because of asthma, symptoms alleviated after a career change. On the other hand, when asthma was mainly the reason for the career change, it was associated with undesirable employment status, work disability or unemployment, compared to full-time workers. The risk for undesirable employment status was 2–6 -fold even after adjusting for age, gender, smoking and professional status.

Earlier studies concerning job changes have mostly dealt with job changes due to respiratory work-related symptoms and work disability or occupational asthma [6, 13–16]. In a study of 196 patients with asthma, as many as 39% believed that asthma had adversely affected their career by causing them to: not pursue a desired career, not get promoted due to absenteeism, change to a worse job, or be perceived as incapable. Changing jobs, work hours or duties was associated with less education, not wanting to work, more comorbidity and more use of asthma medication [16]. In our study, we investigated adults with asthma identified from a general population. Our previous study showed that having frequent asthma symptoms or nightly wake-ups because of asthma is associated with less desirable employment status such as unemployment and work disability. Among individuals with asthma, full-time workers are on average younger, more frequently nonmanual workers, they smoke less and have less asthma symptoms both at work and at leisure time despite of using less asthma medication [11].

In this study, the main purpose was to analyze the career changes adults with asthma had made earlier in their working life and to compare them in current employment status groups. We were able to investigate whether the change had been driven by asthma and furthermore, whether it alleviated or aggravated asthma symptoms. This addresses the question of how much having asthma forces individuals to make changes in their working careers.

Occupation change may be considered a bigger step to take in one’s working career than changing work place or tasks within the same, familiar employer. Changing occupation was less common in our study groups than changing task or work place, but asthma was more often the reason for change in occupation than change in work place. This reflects that the change of occupation is not made on a light basis but it may be that those who change occupation have a more difficult asthma to control. All groups (> 50%) experienced alleviation of asthma symptoms after occupation change and the relief was strongest in the eldest groups, retired and work disability groups.

Of those who had made career changes driven by asthma, the relief of asthma symptoms was most common among full-time workers and more common among other groups than the work disability group. Among the work disability group, aggravation of symptoms after making career changes driven by asthma was more common than in other groups. This suggests that if the subjects can alleviate asthma symptoms by making changes in career, it predicts a favorable career outcome. Still, there is contradiction between the potential beneficial effect of career change on health which is seen as alleviation of asthma symptoms, and the potential deleterious impact of career change at socio-economical level seen in employment status. Even in those who had retired for old age, asthma often seemed to be a reason not to continue in working life and asthma symptoms tended to alleviate in a significant proportion of adults with asthma after retirement.

Full-time workers stand out from the other adults with asthma with having made changes in their earlier working career, whether it is a task change, work place change or occupation change, mostly for other reasons and less frequently driven by their asthma. Our earlier study showed that working full-time was associated with younger age, nonmanual work, less smoking and less symptomatic asthma despite of less asthma medication [11]. Their socio-economical status with younger age and nonmanual work probably also favors changes in their careers because of other reasons than their asthma. It is possible that full-time workers may be overall healthier with less comorbidities which may also enable better flexibility and changes in working life not driven by asthma.

It has been studied earlier that with severe asthma, workers tend to move to jobs involving lower level of exposure [14]. Known as the healthy worker effect, sicker individuals may choose work environments in which exposures are low, they may be excluded from being hired or once hired, they may seek transfer to less exposed jobs or leave work. [17]. In a longitudinal study of adults with occupational asthma, factors associated with individuals staying in their jobs included higher education and income, longer tenure with the company, having children to support and older age [15]. It is common among adults with asthma to change jobs throughout working life due to respiratory problems at work [3].

We studied if asthma was perceived by the subjects to be the reason for retirement and found out that it was significantly more common to retire because of asthma among those with work disability than among those retired for age. However, retirement seemed to similarly relieve asthma symptoms in over 60% of those who had retired regardless of the reason for the retirement. In this study, 90% of the retirement group did not see asthma as a main reason for their retirement, but a great proportion of them reported that asthma symptoms were alleviated after retirement. It may be that alleviation of asthma symptoms is related to lack of job-related stress in addition to less physical strain and exposures. A causal association between chronic psychosocial stress and asthma morbidity has been suggested in studies [18].

In our study, relief of asthma symptoms due to task change was reported by almost half or over half of subjects (48–67%) in all groups which encourages both the employer and occupational health units to support task changes due to asthma. Change of tasks seems to matter and efforts of collaboration between workplace and occupational health units are worthwhile in finding a suitable task, well-being at work and preventing early exit from work.

The strength of our study is the study population which is well representative of asthmatics at working age in Tampere. The response rate was 79%. We recruited all working-age adults with asthma in Tampere city with established asthma and special reimbursement and diagnosis based on lung function criteria. However, as a questionnaire-based study this lacks information on clinical measures and details of treatments on individual level. As a limitation of the study, we had data on all-cause sickness absence and lacked asthma-specific data. One weakness of the study was that in the original questionnaire the subjects were asked if they had made each type of career change after the diagnosis of asthma, but the number of such changes was not asked. It may be that some of the subjects had made several changes of each type, but this cannot be taken into account in the current analysis. Also, there are limitations in the cross-sectional study design. As in all questionnaire-based studies, selection bias is possible and we do not know whether those who answered this questionnaire have e.g. more severe asthma or vice versa. There may also be report bias among those with unfavorable working status. It may be that e.g. asthma was recalled and reported as the reason for a career change more often when the change or the result of the change was undesirable. Our material was collected in year 2000 and some aspects in asthma treatment or working life may have changed. In Finland, our national asthma guidelines and asthma program have stressed active treatment with inhaled corticosteroids (ICS) since early 1990’s [19] and the use of ICS has not changed significantly since the collection of our data. Long-acting beta2-agonists (LABA) were introduced in mid- 1990’s but the use of this class of drugs has probably increased after year 2000 since the introduction of fixed ICS-LABA combinations. Finland has for long had a high level of social security and insurance system that has not significantly changed during the last decades and therefore our results from 2000 are still valid. The economic structure in Finland is typical for countries in Western Europe and has not changed fundamentally after the year 2000.

## Conclusions

Those who were working full time, had most often made changes in their working career, which seem to be supported by younger age, nonmanual work and less asthma symptoms. Being driven to make career changes because of asthma was associated with undesirable employment status, suggesting that symptomatic asthma may accentuate the challenges in working life for adults with asthma. However, career changes especially because of asthma had potential beneficial effect on health seen as alleviation of asthma symptoms after the change. These highlight the importance of proper treatment of asthma, counselling of asthma patients towards applicable area of work or study, and possibly early, proactive support of career changes in maintaining sustainability in working life.
